# Supplementary material for: Research on the evolution and driving forces of the manufacturing industry during the “13th five-year plan” period in Jiangsu province of China based on natural language processing
Source: PLoS One. 2021 Aug 18;16(8):e0256162. doi: 10.1371/journal.pone.0256162 (PMC8372942; doi:10.1371/journal.pone.0256162)
Supplement: S1 Table — (DOCX) [file pone.0256162.s001.docx]

S1 Table: POI keyword d_1_ and industry type c_1_ knowledge base example

| main class | Middle class in manufacturing | Examples of POI keywords | Final classification result |
| --- | --- | --- | --- |
| 1 Agriculture, forestry, animal husbandry, fishery |  | '禽蛋' '禽业' '蔬菜'  ('Poultry eggs' 'poultry industry' 'vegetables') | Non-manufacturing |
| 2 mining industry |  | '采矿业' '煤炭' '开采'  ('Mining industry' 'coal' 'mining') | Non-manufacturing |
| 3 manufacturing | 1 *AF* | '副食品' '提取' '磨制'  ('Non-staple food' 'extraction' 'grinding') | Agricultural and food product processing |
|  | 2 *TC* | '纺织' '棉纺织''印染'  ('Textile' 'Cotton' 'Printing and Dyeing') | Textile clothing |
|  | 3 *WF* | '木材' '竹' '藤'  ('Wood' 'bamboo' 'rattan') | Wood furniture |
|  | 4 *PP* | '体育''娱乐' '用品'  ('Sports' 'Entertainment' 'Supplies') | Pulp and papermaking |
|  | 5 *MC* | '石油' '原油' '煤炭' '日用化工'  ('Petroleum' 'Crude Oil' 'Coal' 'Daily Chemicals') | Metallurgical Chemical Industry |
|  | 6 *PM* | '医' '药''化学药品'  ('Medical' 'medicine' 'chemicals') | Pharmaceutical manufacturing |
|  | 7 *ME* | '测量仪器''电子''电容' '电感'  ('Measuring Instruments' 'Electronics' 'Capacitance' 'Inductance') | Machinery & Electronics |
|  | Unrecognized | — | Unrecognized |
| 4 Electricity, heat, gas and water production and supply |  | '水供应' '电力' '燃气'  ('water supply' 'electricity' 'gas') | Non-manufacturing |
| 5 Construction |  | '房屋建筑' '体育场馆' '城建'  ('House Construction' 'Sports Stadium'  'Urban Construction') | Non-manufacturing |
| 6 Wholesale and retail trade |  | '批发' '进出口'  ('Wholesale' 'Import and Export') | Non-manufacturing |
| 7 Transportation, storage and postal industry |  | '交通运输' '仓储' '邮政业'  ('Transportation' 'Warehouse' 'Postal Industry') | Non-manufacturing |
| 8 Accommodation and Catering Industry |  | '餐饮业' '住宿' '旅游饭店'  ('Restaurant industry' 'accommodation' 'tourist hotel') | Non-manufacturing |
| 9 Information transmission, software and information technology service industry |  | '互联网' '数据' '信息技术'  ('Internet' 'Data' 'Information Technology') | Non-manufacturing |
| 10 Financial Industry |  | '金融' '货币' '金融服务'  ('Finance' 'Currency' 'Financial Services') | Non-manufacturing |
| 11 Real estate industry |  | '开发经营' '物业管理' '房地产'  ('Development and operation' 'Property management' 'Real estate') | Non-manufacturing |
| 12 Leasing and business services |  | '租赁经营' '租房' '租赁'  ('Leasing business' 'Renting a house' 'Leasing') | Non-manufacturing |
| 13 Scientific research and technical services |  | '科学研究' '技术' '服务业'  ('scientific research ' ' technology ' 'service industry ') | Non-manufacturing |
| 14 Water conservancy, environment and public facilities management industry |  | '水利' '环境' '公共' '设施'  ('Water Conservancy' 'Environment' 'Public' 'Facilities') | Non-manufacturing |
| 15 Resident services, repairs and other services |  | '居民' '宠物医院' '修理'  ('Resident' 'Pet Hospital' 'Repair') | Non-manufacturing |
| 16 Education |  | '学前教育' '初等教育' '技能培训'  ('Preschool education' 'Elementary education' 'Skills training') | Non-manufacturing |
| 17 Health and social work |  | '社会工作' '卫生' '医院'  ('Social work' 'Health' 'Hospital') | Non-manufacturing |
| 18 Culture, Sports and Entertainment Industry |  | '期刊' '出版' '音像制品' '彩票'  ('Journal' 'Publishing' 'Audiovisual Products' 'Lottery') | Non-manufacturing |
| Unrecognized |  | — | Unrecognized |
